# An Update on Semaglutide Research: A Bibliometric Analysis and a Literature Review

**DOI:** 10.7759/cureus.46510

**Published:** 2023-10-05

**Authors:** Namrata Dagli, Santosh Kumar, Rahnuma Ahmad, Mahendra Narwaria, Mainul Haque

**Affiliations:** 1 Dentistry, Karnavati Scientific Research Center, Karnavati School of Dentistry, Karnavati University, Gandhinagar, IND; 2 Periodontology and Implantology, Karnavati School of Dentistry, Karnavati University, Gandhinagar, IND; 3 Physiology, Medical College for Women and Hospital, Dhaka, BGD; 4 Bariatric Surgery, Asian Bariatrics Plus Hospital, Ahmedabad, IND; 5 Pharmacology and Therapeutics, National Defence University of Malaysia, Kuala Lumpur, MYS

**Keywords:** hypertension, overweight/obesity, hyperglycemia, type 2 diabetes mellitus, glp-1, glp-1 ra, network visualization, semaglutide, network analysis, bibliometric

## Abstract

This study analyzes the most relevant authors, sources, cooccurrence of keywords, thematic map, and trend topics of the most recent and most cited research papers on semaglutide, a glucagon-like peptide-1 receptor agonist (GLP-1 RA). Also, the content of the 25 most cited papers is summarized. A total of 2995 results appeared in an online electronic search performed on 14 August 2023 in the Scopus database using the term semaglutide. The most recently published 500 articles and most cited 200 documents were selected for bibliometric analysis. Network analysis visualization was conducted with the help of the VOSviewer software (version 1.6.18) (Centre for Science and Technology Studies, Leiden, the Netherlands) and Biblioshiny (it is a shiny application providing a web interface for bibliometrix) (Department of Economics and Statistics, University of Naples Federico II, Naples, Italy).

After excluding duplicates and editorials, the data analysis found that 495 most recent documents were published in 279 journals by 2461 authors, and 200 most cited papers were published in 103 sources by 1241 authors. There is an increasing trend in the number of research papers from 2014 to 2022, with a peak in 2022. The most relevant authors in the most recent semaglutide research papers are Chen and Zhang. The pertinent authors of the most cited research papers on semaglutide are Lingvay and Khunti. The most common keywords used in the most recent and most cited research papers are semaglutide, obesity, diabetes mellitus type 2, glucagon-like peptide-1, glucagon-like peptide-1 receptor agonist, antidiabetic agent, liraglutide, and cardiovascular disease (CVD). The most relevant source is “Diabetes, Obesity and Metabolism” for the research papers on semaglutide. Trend topic analysis suggests that most of the research between 2020 and 2022 on semaglutide was done on non-insulin-dependent diabetes mellitus. The most cited papers provide essential insights into using semaglutide in managing type 2 diabetes mellitus (T2DM), obesity, and related conditions, along with their potential benefits, side effects, and possible mechanisms of action. This analysis highlights that the pharmacological effects of semaglutide extend beyond its role as a glycemic regulator.

## Introduction and background

Traditionally, the emphasis on managing type 2 diabetes mellitus (T2DM) was centered around regulating blood sugar levels. However, contemporary treatment recommendations underscore the significance of a multifaceted approach. This strategy enhances various aspects, including cardiovascular (CV) risk factors, hyperglycemia, overweight/obesity, hypertension, and dyslipidemia [1].

With increasing concerns about the long-term complications of T2DM and the challenges posed by the limited success of existing treatment options, exploring drugs that have the potential to control not only hyperglycemia but also other comorbidities, such as obesity and cardiovascular disease (CVD), has become paramount. On 4 June 2021, the US Food and Drug Administration (FDA) approved the use of semaglutide 2.4 mg for chronic weight management in adults with obesity or overweight with at least one weight-related condition (such as high blood pressure or cholesterol or T2DM) in addition to a reduced calorie diet and increased physical activity [2]. Semaglutide, a glucagon-like peptide-1 receptor agonist (GLP-1 RA), commonly abbreviated as GLP-1 RA, mimics the effects of the hormone glucagon-like peptide-1 (GLP-1), which is naturally produced in the body. GLP-1 helps regulate blood sugar levels by stimulating insulin secretion and suppressing glucagon release [3]. Beyond its ability to stimulate glucose-dependent insulin secretion and suppress glucagon release, semaglutide also reduces hunger food cravings and keeps preference low for food with high-fat content [4,5]. These attributes have prompted investigations into its potential for weight management and CV risk reduction, broadening its scope as a therapeutic agent beyond glycemic control alone.

Furthermore, it is the first GLP-1 RA to be given orally for treating T2DM. Oral administration does not appear to diminish its efficacy in reducing glucose or weight lowering [6]. This comprehensive analysis delves into the burgeoning body of research surrounding semaglutide, aiming to provide an encompassing understanding of the multifaceted nature of semaglutide-provoked pharmacological effects.

This article aims to provide a comprehensive analysis of the research on semaglutide. It seeks to contribute to the ongoing discourse on the role of semaglutide in reshaping the therapeutic landscape for T2DM and potentially beyond. This study separately analyzed the most contributing authors, most relevant journals, cooccurrences of keywords, thematic map, and trend topics for the most recent and most cited papers on semaglutide. The study also includes a literature review of the 25 most cited documents on semaglutide.

## Review

Materials and methods

Search Strategy and Study Selection Process

An online electronic search was performed on 14 August 2023 in the Scopus database to identify the research done on semaglutide. The search term used was semaglutide. No filter was applied for the type of articles, species, language, gender, journal, age, or publication date in the database. The most recent 500 papers were selected for the bibliometric analysis. The most cited research papers were identified by arranging the research papers according to the number of citations in the Scopus database, and the most cited 200 research papers were selected for bibliometric analysis.

The study selection process flow chart was generated according to the Preferred Reporting Items for Systematic Reviews and Meta-Analyses (PRISMA) guidelines [7]. The data was exported from the Scopus database and saved as an Excel file. Three duplicates were identified and removed. Two editorials were also removed.

Data Analysis

Network analysis and visualization were performed using the VOSviewer software (version 1.6.18) (Centre for Science and Technology Studies, Leiden, the Netherlands) [8], and Biblioshiny (Department of Economics and Statistics, University of Naples Federico II, Naples, Italy), a web-based application for comprehensive science mapping analysis [9]. VOSviewer is a software tool for constructing and visualizing bibliometric networks. The analysis of author productivity and core sources was done by Biblioshiny.

Review

The content of the top 20 most cited papers was analyzed and summarized. Our review included the following stages: identifying the research question and relevant studies, study selection, charting the data, collating, summarizing, and reporting the results [10].

Results

The Search Results for the Most Recent Research Papers

**Figure 1:**
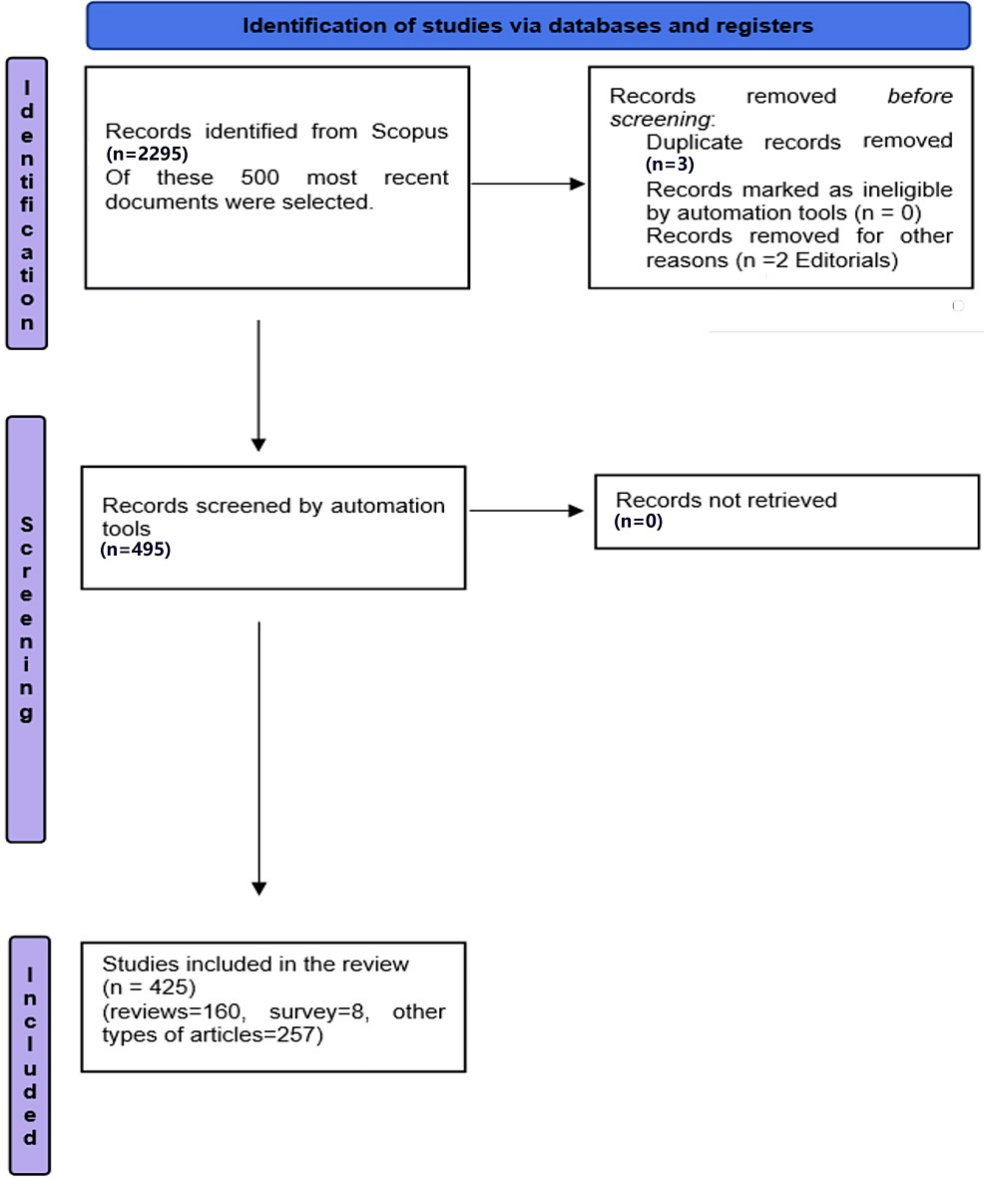
A flow chart depicting the process of selecting studies for the most recent and most cited research papers

A total of 2995 results appeared from an online search in the Scopus database without applying any filter. Of these, 38.7% were reviews, 7.3% were notes, 3.9% were letters, 3.6% were editorials, 1.2% were errata, 1% were short surveys, 0.9% were book chapters, 0.4% were conference papers, and 43% were other articles. Five hundred most recent research papers were selected, of which 495 articles were left after removing duplicates and editorials. The total number of sources of research papers on semaglutide was 279, and the total number of authors was 2461. The type of documents selected for the analysis were articles, reviews, and short surveys, whereas book chapters, editorials, errata, letters, and notes were excluded from the analysis. After excluding the articles according to the above criteria, the total number of documents left was 425, published in 250 journals by 2295 authors. Out of 425 papers, 160 are reviews, eight are short surveys, and 257 are other articles (Figure 1). The average citation per document is 1.25. Single-authored research papers were 18, and co-authors per document were 6.3. Single countries publish all 425 research papers, and no collaboration between countries is found.

Publishing Trend of Research Papers on Semaglutide

**Figure 2:**
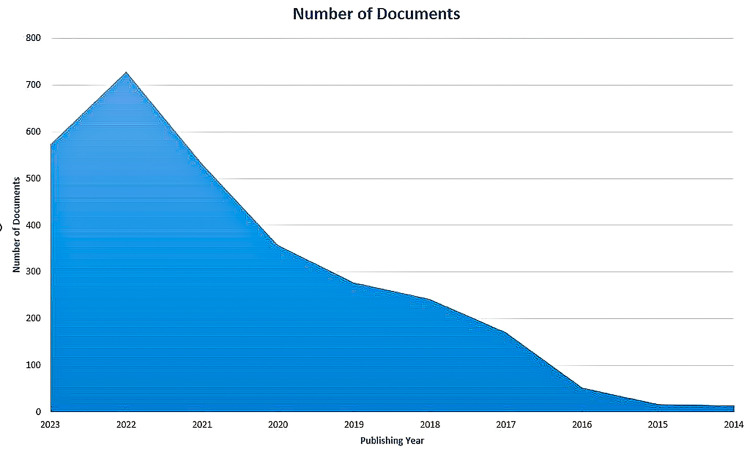
The publishing trend of research papers on semaglutide

Figure 2 shows an increasing trend in research papers from 2014 to 2022, with a rapid increase from 2020 to 2022. The maximum number of research papers is published in 2022.

Most Relevant Authors and Authors’ Productivity Analysis for the Most Recent Research Papers

**Figure 3:**
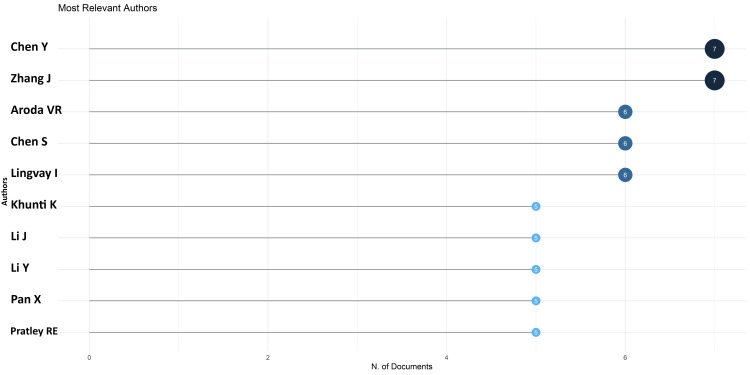
Most relevant authors in the most recent research papers on semaglutide

The most pertinent authors for the most recent semaglutide research papers are Chen and Zhang, with seven papers each (Figure 3). The 10 most relevant authors published 47 research papers (11%) out of 425.

**Figure 4:**
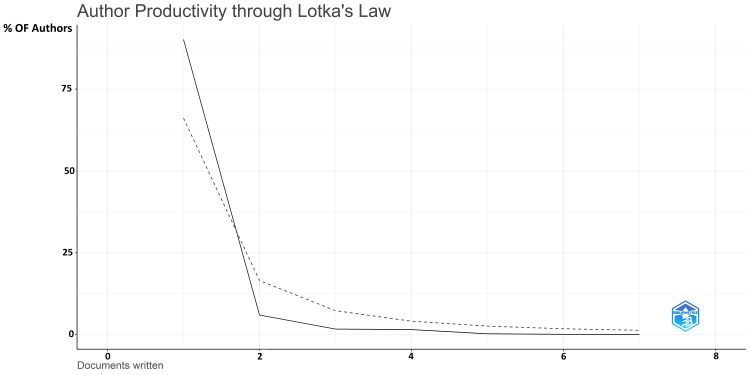
Author productivity analysis through Lotka’s law The shaded line represents the expected values

The authors’ productivity analysis was done using Lotka’s law in Biblioshiny. Lotka’s law, also known as Lotka’s law of scientific productivity, is an empirical principle that describes the distribution of scientific productivity among researchers in each field. It was formulated by Alfred J. Lotka, an American mathematician and statistician, in the early 20th century. Lotka’s law is often used to model the distribution of scientific publications [11]. The number of publications is plotted on the X-axis, and the percentage of authors is on the Y-axis (Figure 4). The graph clearly shows that most of the authors have published one document.

Most Relevant Sources

**Figure 5:**
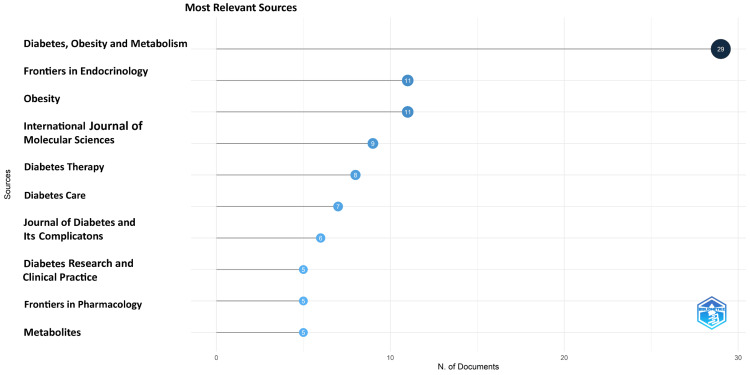
Most relevant sources of the most recent research papers on semaglutide

The most relevant source for the most recent research papers on semaglutide is “Diabetes, Obesity and Metabolism,” with 29 research papers on semaglutide that alone contributed to almost 30% of the papers published by all 10 most relevant journals (i.e., 96). The other important sources are Frontiers in Endocrinology and Obesity, with 11 (11.5%) research papers each (Figure 5). The graph suggests that most journals have published less than 10 documents.

Keyword Cooccurrence Analysis

**Figure 6:**
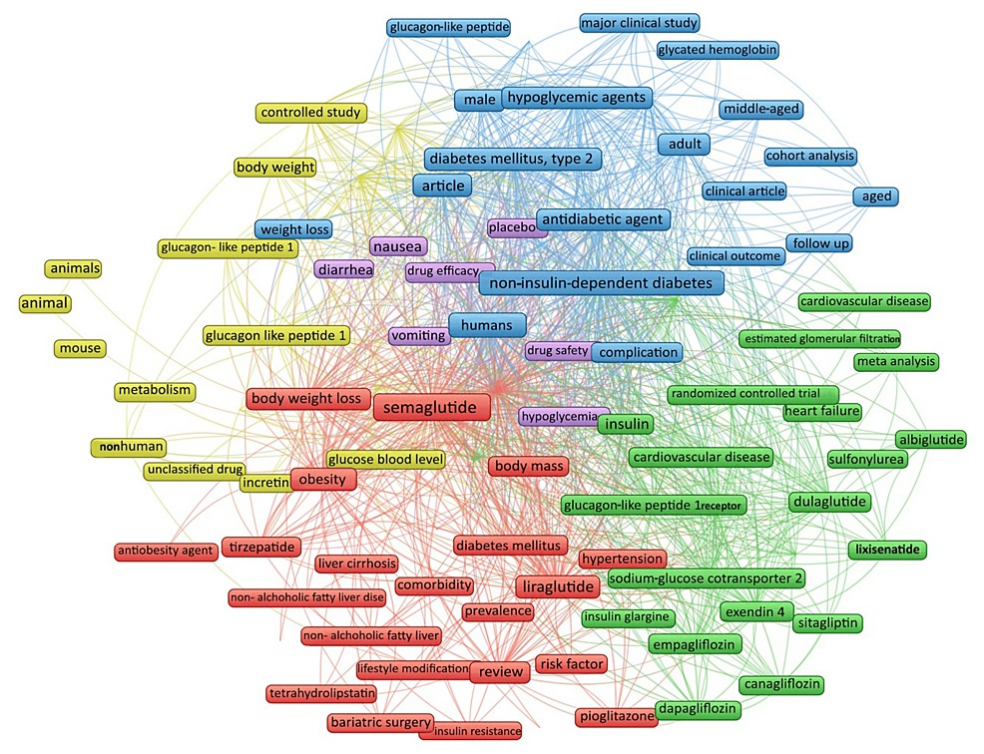
Network analysis of keyword cooccurrence in the most recent research papers on semaglutide The font size represents the frequency of keywords. Clusters 1, 2, 3, 4, and 5 are represented by red, green, blue, yellow, and purple, respectively

The cooccurrence analysis of keywords used in most recent research papers was done using VOSviewer. The unit of analysis was all keywords. We selected the keywords with a minimum occurrence of 30. Of 5387 keywords, 101 met the criteria. For each of the 101 keywords, the total strength of the cooccurrence links with other keywords was calculated, and the keywords with the greatest total link strength were selected. A total of five clusters appeared in the network analysis with 4877 links and 93762 total link strength. The items in clusters 1, 2, 3, 4, and 5 are 27, 26, 24, 16 and eight, respectively (Figure 6). The most common keywords are semaglutide, obesity, diabetes mellitus type 2, and GLP-1.

A thematic map is a visual representation of the thematic structure of a research field. It is divided into four categories: motor themes, basic and transversal themes, highly developed and isolated themes, and emerging or declining themes [12].

**Figure 7:**
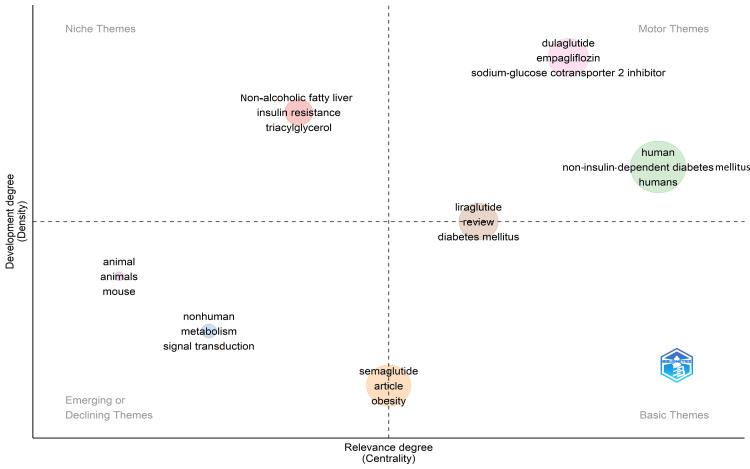
Thematic map (by Biblioshiny) for the most recent research papers on semaglutide It is based on density (Y-axis) and centrality (X-axis). The centrality measures the importance of the selected theme, and the density measures the development of the chosen theme

In bibliometric analysis, motor themes refer to the most important research topics that drive the research forward. In this thematic map, dulaglutide, empagliflozin, liraglutide, sodium-glucose cotransporter 2 inhibitor, humans, and non-insulin-dependent diabetes mellitus are the motor terms. Niche theme topics not well connected to other issues in the field yet important are triacylglycerol, insulin resistance, and non-alcoholic fatty liver. Emerging themes and issues gaining importance in the area but have not yet become mainstream are nonhuman, animal, mouse, metabolism, and single transduction. Basic themes well established in the field are diabetes mellitus, semaglutide, and obesity. The thematic map suggests that obesity, liraglutide, and diabetes mellitus are the most important and developed themes in research papers on semaglutide (Figure 7).

Analysis of most cited research papers on semaglutide

The Search Results of the Most Cited Articles

A total of 200 most cited papers were published in 103 sources by 1241 authors between the years 2020 and 2023. The type of documents selected for the analysis was articles and reviews, whereas book chapters, conference papers, and notes were excluded from the analysis. After excluding the articles according to the above criteria, the total number of documents left was 197, published in 101 journals by 1236 authors (Figure 1). Out of 197 papers, 112 are reviews, and 85 are other articles. The average citation per document is 94.46. Single-authored documents were six, and co-authors per document were 7.54. No collaboration between any country is found. The annual growth rate is 76.39%. A total of 3401 keywords were identified.

Analysis of Authors of the Most Cited Research Papers

**Figure 8:**
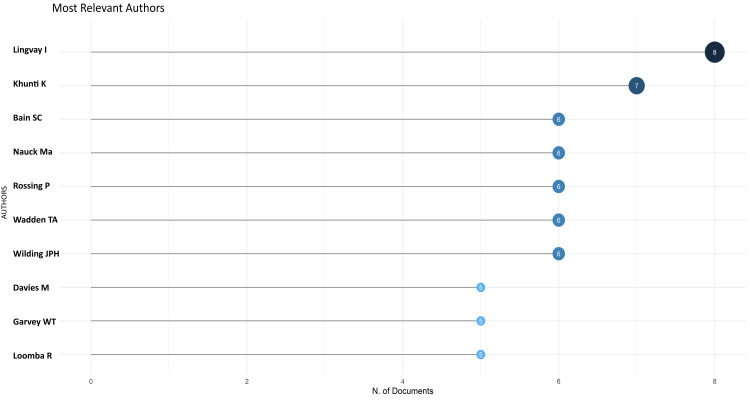
Most relevant authors in the most cited research papers on semaglutide

The most relevant authors for the most cited publications are listed in Figure 8. The most pertinent authors of the most cited research papers on semaglutide are Lingvay and Khunti, with eight and seven papers, respectively. The 10 most relevant authors published 60 research papers (30.5%) out of 197 (Figure 8).

Analysis of Sources

**Figure 9:**
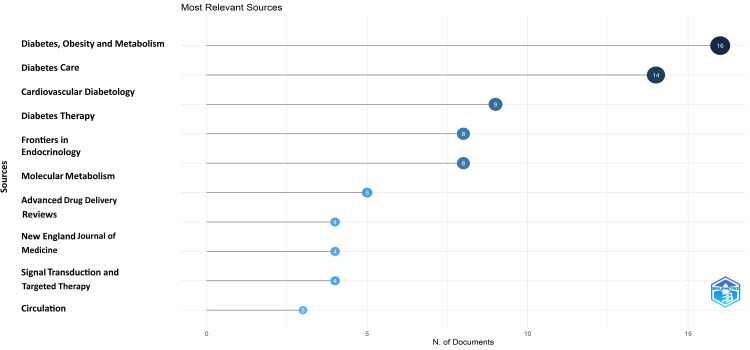
Most relevant sources of the most cited research papers

**Figure 10:**
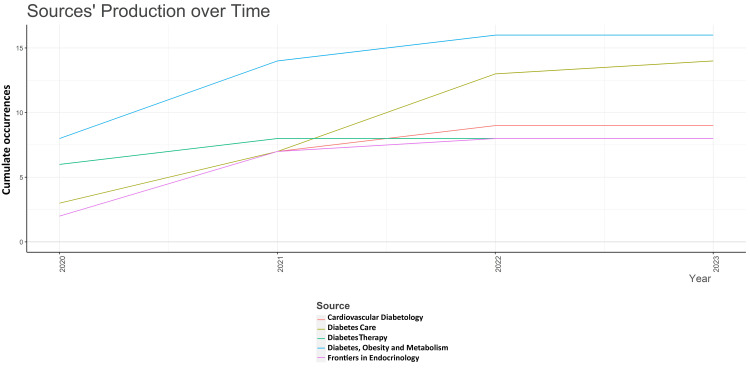
Sources’ production over time for the most cited research papers

**Figure 11:**
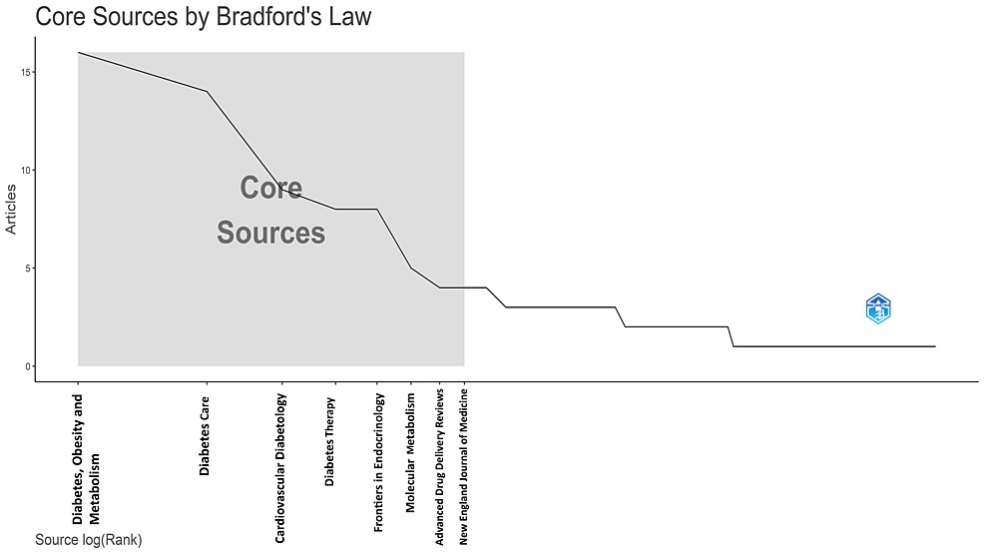
Core sources by Bradford’s law for the most cited research papers on semaglutide

The most relevant source is “Diabetes, Obesity and Metabolism” and “Diabetes Care,” with 16 and 14 research papers on semaglutide, respectively. These two contributed to almost 40% of the most cited research papers published by all 10 most relevant journals (i.e., 75), and “Diabetes, Obesity and Metabolism” alone contributed 21.3% of them (Figure 9). The graph suggests that five of the most relevant 10 sources (50%) of the most cited papers have published more than five research papers. The number of articles published in various sources over time and the core sources for the most cited research papers are presented in Figure 10 and Figure 11.

The core sources are analyzed according to Bradford’s law in Biblioshiny for the most cited research papers (Figure 11). The shaded area represents the number of articles published by core sources. Bradford’s law is often used in bibliometrics, which involves quantitatively analyzing scholarly publications and their bibliographic characteristics. The law helps understand the concentration of relevant literature and determine the core journals covering a particular subject area. The law suggests that the number of journal articles on a subject follows a geometric progression. The nucleus consists of a few core journals that publish a significant number of articles on the topic, followed by a more substantial number of journals in the first zone, an even more significant number in the second zone, and so on. The relationship between the number of journals and articles in each zone follows a predictable pattern [13].

Keywords and Trend: Topic Analysis

**Figure 12:**
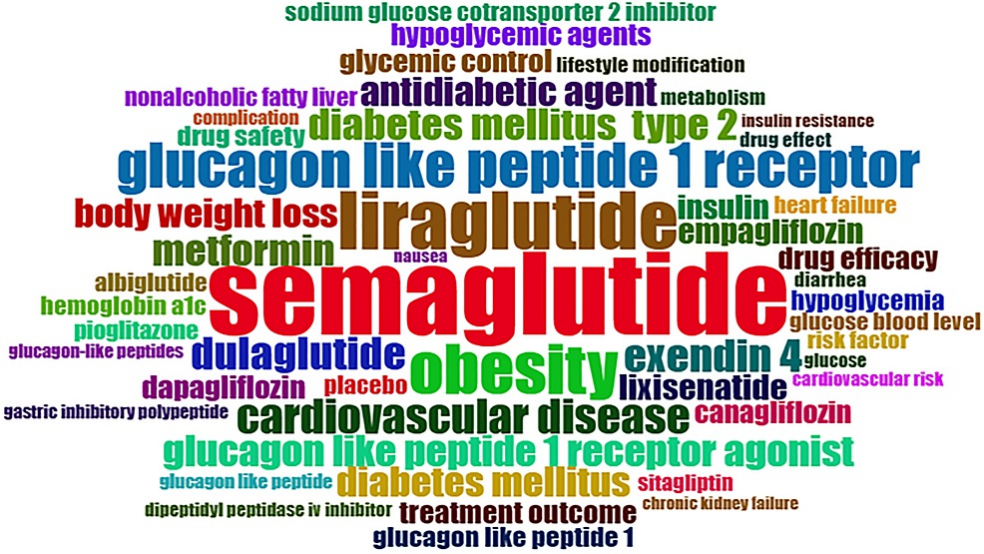
The word cloud shows the keywords for the most cited research papers on semaglutide, and the size represents the frequency of their use

The word cloud for the most cited research papers on semaglutide shows the most frequently used keywords in research papers. The following 23 keywords, not specific to the research subject, were removed so that subject-specific keywords can be seen more clearly in an image: human, humans, adult, female, male, review, RCT, controlled study, aged, nonhuman, systematic review, meta-analysis, cohort analysis, clinical articles, mouse, animal, animals, randomized controlled study, randomized controlled trials, middle-aged, priority journal, article, and semaglutide. The following two synonymous terms were also merged to obtain the actual frequency of use in research papers: glucagon-like peptide 1 receptor and glucagon-like peptide-1 receptor (Figure 12). The most common keywords used in the most recent research papers are semaglutide, obesity, T2DM, diabetes mellitus type 2 and glucagon-like peptide-1, glucagon-like peptide-1 receptor agonist, antidiabetic agent, liraglutide, and cardiovascular disease.

**Figure 13:**
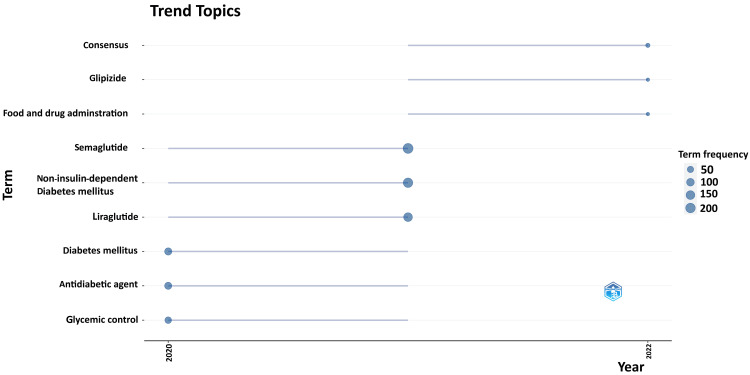
Trend topic analysis of the most cited research papers

The trend topic analysis graph shows the frequency of keywords used in research papers on semaglutide. The most frequently used terms were semaglutide, non-insulin-dependent diabetes mellitus, and liraglutide between 2020 and 2022. The least used but most recent terms were glipizide, Food and Drug Administration, and consensus. Diabetes mellitus, antidiabetic agents, and glycemic control were used more commonly in 2020 (Figure 13). This suggests that most of the research between 2020 and 2022 on semaglutide was centered on non-insulin-dependent diabetes mellitus and glycemic control. The effects of other drugs, liraglutide and glipizide, were also studied on diabetes along with semaglutide.

Results of the Review of the Most Cited Papers

Of the 25 most cited research papers, six are reviews, and others are guidelines and clinical trials. Ten articles are guidelines provided by the American Diabetes Association (ADA), one is guidelines provided by the European Association for the Study of Diabetes (EASD), one is guidelines provided by the American Association of Clinical Endocrinology Clinical Practice Guideline for the Diagnosis and Management of Nonalcoholic Fatty Liver Disease, and two are consensus reports. A total of 16 articles are related to the effect of semaglutide on diabetes, five related to obesity, two related to non-alcoholic fatty liver, and two related to both diabetes and obesity.

The most cited paper has been cited 2302 times. It is based on the 2019 guidelines by the European Society of Cardiology on diabetes, pre-diabetes, and cardiovascular diseases in collaboration with the EASD [14]. The second most cited article has been cited 824 times. This double-blind clinical trial included 1961 adults with a body mass index of 30 or greater without diabetes. Semaglutide 2.4 mg or placebo was given once weekly for 68 weeks via the subcutaneous route. Cardiometabolic risk factors and participant-reported physical functioning were improved more in the semaglutide group from baseline than in the placebo group [15].

“Pharmacologic Approaches to Glycemic Treatment: Standards of Medical Care in Diabetes,“ published in 2020 [16] and 2021 [17], have been cited by 730 and 703, respectively. The guidelines by ADA suggest that in patients with established cardiovascular disease, kidney disease, or heart failure, sodium-glucose cotransporter 2 inhibitors or glucagon-like peptide-1 RA should be considered regardless of glycated hemoglobin (HbA1c) levels. GLP-1 RA is preferred over insulin when stronger glucose lowering is needed in patients with type 2 diabetes. However, cost and tolerability issues are essential considerations in GLP-1 RA use [16-19]. ADA also suggests that GLP-1 RA reduces the risk of atherosclerotic major adverse cardiovascular events to a comparable degree in patients with T2DM and established atherosclerotic cardiovascular disease [20,21]. Semaglutide minimizes the risk of new or worsening nephropathy in T2DM patients [22,23] and does not require dose adjustment for reduced estimated glomerular filtration rate [24]. A consensus report by the American Diabetes Association (ADA) and the European Association for the Study of Diabetes (EASD) on managing T2DM mentioned that semaglutide is highly effective for weight management in people with type 2 diabetes [25]. The American Association of Clinical Endocrinology Clinical Practice Guideline for the Diagnosis and Management of Nonalcoholic Fatty Liver Disease recommends that clinicians must consider obesity pharmacotherapy with a preference for semaglutide 2.4 mg/week as adjunctive therapy to lifestyle modification for individuals with obesity and non-alcoholic fatty liver disease (NAFLD) or non-alcoholic steatohepatitis (NASH) to promote cardiometabolic health and treat or prevent T2DM, cardiovascular diseases (CVDs), and other end-stage manifestations of obesity. It reduces myocardial infarction and stroke events and is proven to reverse steatohepatitis in persons with or without diabetes [26].

In a phase 3 trial, 1879 patients were randomly assigned in a 1:1:1:1 ratio to receive tirzepatide at a dose of 5 mg, 10 mg, or 15 mg or semaglutide at a dose of 1 mg for 40 weeks. The study concluded that tirzepatide is non-inferior and superior to semaglutide in reducing weight and mean glycated hemoglobin level; however, more subjects experienced adverse events [27]. Another double-anonymized phase 2 trial done on 320 patients for 72 weeks involving patients with biopsy-confirmed NASH and liver fibrosis is cited by 564 research papers. Patients were randomly assigned, in a 3:3:3:1:1:1 ratio, to receive once-daily subcutaneous semaglutide at a dose of 0.1, 0.2, or 0.4 mg or a corresponding placebo. Novo Nordisk funded this study. This study demonstrated that NASH resolution occurred in a significantly higher number of patients than placebo; however, between-group difference was not significant for improving the fibrosis stage [28]. One more randomized study double-masked phase 3a trial that compared semaglutide to placebo for weight maintenance along with lifestyle modification in 902 adults with overweight or obesity after administering semaglutide 2.4 mg weekly via subcutaneous route for 20 weeks demonstrated that semaglutide resulted in continued weight loss over the following 48 weeks. The duration of the trial was 68 weeks [29]. Adults with a body mass index of at least 30 (or 27 with one weight-related comorbidity) and without diabetes were included. Another phase 3 randomized trial was conducted at 41 sites in the United States between 2018 and 2020. This study population was 611 adults without diabetes and with either overweight plus at least one comorbidity or obesity for 68 weeks. This study concluded that the subcutaneous administration of semaglutide 2.4 mg per week, compared to placebo, caused significantly more weight loss when used as an adjunct to intensive behavioral therapy and an initial low-calorie diet [30].

One review performed by Bornstein et al. cited 560 research papers that provided recommendations for the practical management of patients with diabetes and COVID-19 disease or at risk for metabolic disease based on the published literature and guidelines by the WHO, American Diabetes Association, and US Centers for Disease Control and Prevention for the management of COVID-19 [31]. Another review by Brown et al. highlighted that there is compelling data on the benefits of these drugs for a range of other clinical indications even without type 2 diabetes, including for GLP-1 RA in patients with obesity and overweight with weight-related comorbidities. However, GLP-1 RA is contraindicated in those with a history of medullary thyroid cancer and is used cautiously in patients with a history of pancreatitis of a known cause [32].

A review done by Nauck et al. suggests that GLP-1 RA lowers body weight by influencing the central nervous system and shares common mechanisms of action, which include enhancing insulin in response to hyperglycemia, suppressing glucagon secretion in hyperglycemia or euglycemia, slowing gastric emptying to prevent large post-meal spikes in blood sugar, and reducing calorie intake. Recently, an oral formulation of semaglutide (once daily) has been approved. Oral formulation possesses equivalent clinical effectiveness when compared to the once-weekly subcutaneous preparation. Furthermore, it has similar effectiveness for HbA1c reduction with additional weight reduction and no intrinsic risk of hypoglycemic episodes. It is recommended as the preferred first injectable glucose-lowering therapy for T2DM, even before insulin treatment. Glucagon-like peptide-1 receptor (GLP-1 R) also reduces cardiovascular events, such as myocardial infarction, stroke, and associated mortality [3]. It has been reported that semaglutide causes a sustained reduction in body weight of approximately 0.5 kg per week [33].

According to the summary on T2DM management by the American Association of Clinical Endocrinology and the American College of Endocrinology, GLP-1 RA based on exendin 4 has been proven safe in cardiovascular disease. Still, they have not been shown to confer cardiovascular benefits. The risk of hypoglycemia with GLP-1 RA is low, and they reduce fluctuations in both fasting and postprandial glucose levels by stimulating glucose-dependent insulin secretion and suppressing glucagon secretion. GLP-1 RA should not be used in patients with a personal or family history of medullary thyroid carcinoma or those with multiple endocrine neoplasia syndrome type 2. No dose adjustment is required for semaglutide in chronic kidney disease, although renal function should be monitored in patients reporting severe adverse gastrointestinal reactions [34]. According to a review, the proper understanding of the NAFLD spectrum, as a continuum from obesity to metabolic syndrome and diabetes, is essential for the early identification and the establishment of targeted treatment [35]. A meta-analysis concluded that GLP-1 RA, regardless of structural homology, reduced the risk of individual major adverse cardiovascular event components, all-cause mortality, hospital admission for heart failure, and worsening kidney function in patients with type 2 diabetes [36].

An animal study by Gabery et al. demonstrated that GLP-1 RA semaglutide reduces body weight through direct effects in the hypothalamus and the hindbrain. Semaglutide directly accessed the brainstem, septal nucleus, and hypothalamus and interacted with the brain through the circumventricular organs and several select sites adjacent to the ventricles of rats. Semaglutide interacts with 10 brain areas: hindbrain areas directly with GLP-1 R interaction and secondary areas without direct GLP-1 RA interaction, such as the lateral parabrachial nucleus. The automated analysis of semaglutide revealed that activation may involve meal termination controlled by neurons in the lateral parabrachial nucleus. The transcriptomic analysis of microdissected brain areas from semaglutide-treated rats showed the upregulation of prolactin-releasing hormone and tyrosine hydroxylase in the area postrema. This study suggests that semaglutide lowers body weight by direct interaction with diverse GLP-1 R populations and by directly and indirectly affecting the activity of neural pathways involved in food intake, reward, and energy expenditure [37]. The common side effects reported in clinical trials are nausea, constipation, diarrhea, and vomiting. Two clinical trials reported that subjects discontinued treatment due to gastrointestinal events [15,30]. One study also reported neoplasms in 15% of the patients receiving semaglutide compared to 8% in those who received placebo. However, no relationship with any specific organs was noted [28]. The summary of the 25 most cited articles is presented in Table 1 [3,14-37].

**Table 1:** Summary of the most cited papers on semaglutide in Scopus database ADA, American Diabetes Association; EASD, European Association for the Study of Diabetes; GLP-1, glucagon-like peptide-1; GLP-1 RA, glucagon-like peptide-1 receptor agonists; ASCVD, atherosclerotic cardiovascular disease; SGLT2, sodium-glucose cotransporter 2; BW, body weight; CV, cardiovascular; CVD, cardiovascular disease; T2DM, type 2 diabetes mellitus; NAFLD, non-alcoholic fatty liver disease; NASH, non-alcoholic steatohepatitis; MACE, major adverse cardiovascular events; HbA1c, glycated hemoglobin; SF-36, 36-Item Short Form Health Survey; ASCVE, atherosclerotic cardiovascular events

Authors	Title	Source title	Citation	Results	Document type
Cosentino et al., 2020 [14]	2019 ESC Guidelines on diabetes, pre-diabetes, and cardiovascular diseases developed in collaboration with the EASD: The Task Force for diabetes, pre-diabetes, and cardiovascular diseases of the European Society of Cardiology (ESC) and the European Association for the Study of Diabetes (EASD)	European Heart Journal	2302	Information not available.	Article
Wilding et al., 2021 [15]	Once-Weekly Semaglutide in Adults with Overweight or Obesity	New England Journal of Medicine	824	A weekly dosage of 2.4 mg of semaglutide and lifestyle intervention significantly decreased body weight. The mean change from baseline to week 68 was significantly higher in the semaglutide group (14.9%) as compared to placebo (2.4%) (P<0.001). The most frequent adverse events were mild to moderate nausea and diarrhea, which gradually diminished. A greater number of participants in the semaglutide group (4.5%) than in the placebo group (0.8%) discontinued the study due to gastrointestinal events.	Randomized clinical trial
American Diabetes Association, 2020 [16]	Pharmacologic Approaches to Glycemic Treatment: Standards of Medical Care in Diabetes-2020	Diabetes Care	730	This paper includes the ADA’s current recommendations for diabetes care, general treatment goals and guidelines, and quality of care evaluation tools. The guidelines recommend that in patients with established cardiovascular disease, kidney disease, or heart failure, SGLT2 inhibitors or GLP-1 RA should be considered regardless of HbA1c levels.	Article
American Diabetes Association, 2021 [17]	Pharmacologic Approaches to Glycemic Treatment: Standards of Medical Care in Diabetes-2021	Diabetes Care	703	This paper includes the ADA’s current recommendations for diabetes care, general treatment goals and guidelines, and quality of care evaluation tools. The guidelines recommend that in patients with established cardiovascular disease, kidney disease, or heart failure, SGLT2 inhibitors or GLP-1 RA should be considered regardless of HbA1c levels.	Article
Newsome et al., 2021 [28]	A Placebo-Controlled Trial of Subcutaneous Semaglutide in Nonalcoholic Steatohepatitis	New England Journal of Medicine	564	The NASH resolution was noted without worsening of fibrosis in 40% of subjects in the 0.1 mg group, 36% in the 0.2 mg group, 59% in the 0.4 mg group, and 17% in the placebo group. The difference was significant between semaglutide 0.4 mg and the placebo group. An improvement in the fibrosis stage occurred in 43% of the patients in the 0.4 mg group and 33% of the patients in the placebo group (P=0.48). The average percent weight loss was 13% in the 0.4 mg group and 1% in the placebo group. The incidence of nausea, constipation, and vomiting was higher in the 0.4 mg group than in the placebo group. Malignant neoplasms were noted in three subjects in the semaglutide group (1%) but not in the placebo group. Overall, neoplasms were reported in 15% of the patients in the semaglutide group and 8% in the placebo group.	Phase 2 clinical trial
Bornstein et al., 2020 [31]	Practical recommendations for the management of diabetes in patients with COVID-19	The Lancet Diabetes & Endocrinology	560	Provides brief insight into potential mechanistic links between the novel coronavirus infection and diabetes, presents practical management recommendations, and elaborates on the differential needs of several patient groups.	Review
American Diabetes Association Professional Practice Committee, 2022 [18]	Pharmacologic Approaches to Glycemic Treatment: Standards of Medical Care in Diabetes-2022	Diabetes Care	423	The guidelines recommend that in patients with established cardiovascular disease, kidney disease, or heart failure, SGT2 inhibitors or GLP-1 RA should be considered regardless of HbA1c levels.	Article
American Diabetes Association, 2020 [21]	Cardiovascular Disease and Risk Management: Standards of Medical Care in Diabetes-2020	Diabetes Care	409	GLP-1 receptor agonists and SGLT2 inhibitors reduce the risk of atherosclerotic major adverse cardiovascular events to a comparable degree in patients with type 2 diabetes and established ASCVD.	Article
Frías et al., 2021 [27]	Tirzepatide versus Semaglutide Once Weekly in Patients with Type 2 Diabetes	New England Journal of Medicine	389	Tirzepatide at all doses was non-inferior and superior to semaglutide. BW reduction was significantly more substantial with tirzepatide than semaglutide (P<0.001). The most common adverse events were gastrointestinal and were primarily mild to moderate in severity in the tirzepatide and semaglutide groups (nausea, 17%-22% and 18%; diarrhea, 13%-16% and 12%; and vomiting, 6%-10% and 8%, respectively). Of the patients who received tirzepatide, hypoglycemia (blood glucose level: <54 mg per deciliter) was reported in 0.6% (5 mg group), 0.2% (10 mg group), and 1.7% (15 mg group); hypoglycemia was reported in 0.4% of those who received semaglutide. Serious adverse events were reported in 5%-7% of the patients receiving tirzepatide and 3% receiving semaglutide.	Phase 3 clinical trial
Buse et al., 2020 [19]	2019 Update to: Management of Hyperglycemia in Type 2 Diabetes, 2018. A Consensus Report by the American Diabetes Association (ADA) and the European Association for the Study of Diabetes (EASD)	Diabetes Care	352	The ADA and the EASD have briefly updated their 2018 recommendations on managing hyperglycemia based on findings from large CV outcome trials published in 2019. Significant updates are the following: (1) the decision to treat high-risk individuals with a GLP-1 receptor agonist or SGLT2 inhibitor to reduce MACE, hospitalization for heart failure, cardiovascular death, or chronic kidney disease (CKD) progression should be considered independently of baseline HbA1c or individualized HbA1c target; (2) GLP-1 receptor agonists can also be considered in patients with type 2 diabetes without established CVD but with the presence of specific indicators of high risk.	Article
Garber et al., 2020 [34]	Consensus Statement by the American Association of Clinical Endocrinologists and American College of Endocrinology on the Comprehensive Type 2 Diabetes Management Algorithm – 2020 Executive Summary	Endocrine Practice	343	This document represents the summary of type 2 diabetes management proposed by the American Association of Clinical Endocrinology and the American College of Endocrinology. According to this paper, the risk of hypoglycemia with GLP-1 receptor agonists is low, and they reduce fluctuations in both fasting and postprandial glucose levels. No dose adjustment is required for semaglutide in chronic kidney disease, but monitoring is recommended.	Review
Nauck et al., 2021 [3]	GLP-1 receptor agonists in the treatment of type 2 diabetes – state-of-the-art	Molecular Metabolism	307	Semaglutide has more profound effects on overnight and fasting plasma glucose and HbA1c, both on a background of oral glucose-lowering agents and in combination with basal insulin. The influence on gastric emptying reduces over time. Semaglutide is characterized by a greater efficacy in lowering plasma glucose and body weight. GLP-1 RAs can effectively prevent CV adverse events and renal complications associated with T2DM.	Review
American Diabetes Association, 2020 [22]	Microvascular Complications and Foot Care: Standards of Medical Care in Diabetes-2020	Diabetes Care	304	This paper includes the ADA’s current recommendations for diabetes care, general treatment goals and guidelines, and quality of care evaluation tools. According to this paper, semaglutide reduces the risk of new or worsening nephropathy in T2DM patients.	Article
Sattar et al., 2021 [36]	Cardiovascular, mortality, and kidney outcomes with GLP-1 receptor agonists in patients with type 2 diabetes: a systematic review and meta-analysis of randomised trials	The Lancet Diabetes & Endocrinology	273	GLP-1 RA reduced MACE by 14% (P<0.0001), with no significant heterogeneity across GLP-1 RA structural homology or eight other examined subgroups. GLP-1 RA reduced all-cause mortality by 12% (P=0.0001), hospital admission for heart failure by 11% (P=0.013), and the composite kidney outcome by 21% (P<0.0001), with no increase in the risk of severe hypoglycemia, retinopathy, or pancreatic adverse effects.	Systematic review and meta-analysis
Rubino et al., 2021 [29]	Effect of Continued Weekly Subcutaneous Semaglutide vs Placebo on Weight Loss Maintenance in Adults With Overweight or Obesity: The STEP 4 Randomized Clinical Trial	Journal of the American Medical Association	269	The mean body weight change from week 20 to week 68 was significantly (P<0.001) reduced in the semaglutide group (7.9%) when compared to the group with a switch to placebo (6.9%). Waist circumference, systolic blood pressure, and SF-36 physical functioning score also improved with continued subcutaneous semaglutide (P<0.001). Gastrointestinal events were experienced by 49% of participants who remained on semaglutide and 26% with placebo. The number of participants who discontinued treatment due to adverse events with continued semaglutide (2.4%) and placebo (2.2%) was similar.	Randomized phase 3 clinical trial
Wadden et al., 2021 [30]	Effect of Subcutaneous Semaglutide vs Placebo as an Adjunct to Intensive Behavioral Therapy on Body Weight in Adults with Overweight or Obesity: The STEP 3 Randomized Clinical Trial	Journal of the American Medical Association	255	The estimated mean body weight change from baseline was 16.0% for semaglutide while 5.7% for placebo after 68 weeks.	Randomized double-blind trial
American Diabetes Association Professional Practice Committee, 2022 [20]	Cardiovascular Disease and Risk Management: Standards of Medical Care in Diabetes-2022	Diabetes Care	226	This paper includes the ADA’s current recommendations for diabetes care, treatment goals and guidelines, and quality of care evaluation tools. GLP-1 receptor agonists and SGLT2 inhibitors reduce the risk of ASCVE to a comparable degree in patients with type 2 diabetes and established ASCVD.	Article
American Diabetes Association, 2021 [23]	Microvascular Complications and Foot Care: Standards of Medical Care in Diabetes-2021	Diabetes Care	215	The paper includes the ADA’s recommendations for diabetes care, treatment goals and guidelines, and quality of care evaluation tools. According to this paper, semaglutide reduces the risk of new or worsening nephropathy in T2DM patients.	Article
Godoy-Matos et al., 2020 [35]	NAFLD as a continuum: From obesity to metabolic syndrome and diabetes	Diabetology & Metabolic Syndrome	209	This review focuses on the clinical and pathophysiological connections between NAFLD, insulin resistance, and type 2 diabetes. Current available GLP-1 RA liraglutide and semaglutide might improve obesity and T2DM that impact each stage of NAFLD. Understanding the NAFLD spectrum correctly, which encompasses a range from obesity to metabolic syndrome and diabetes, could aid in the early detection and implementation of tailored therapeutic approaches.	Review
Müller et al., 2022 [33]	Anti-obesity drug discovery: Advances and challenges	Nature Reviews Drug Discovery	187	Semaglutide at a dose of 2.4 mg lowers mean body weight to ~15% after 68 weeks of treatment (relative to ~2.4% in placebo controls). The drug is generally well tolerated, primarily in nausea, diarrhea, vomiting, and constipation.	Review
Gabery et al., 2020 [37]	Semaglutide lowers body weight in rodents via distributed neural pathways	JCI Insight	187	Semaglutide reduces weight, glucose levels, and CV risk in individuals with T2DM. Preclinical studies with a mechanical approach indicate that weight loss occurs due to the brain’s activation of GLP-1 receptors (GLP-1 Rs). The results presented in this context demonstrate that semaglutide altered food preferences, decreased food consumption, and caused weight loss without reducing energy expenditure.	Article
Davies et al., 2022 [25]	Management of Hyperglycemia in Type 2 Diabetes, 2022. A Consensus Report by the American Diabetes Association (ADA) and the European Association for the Study of Diabetes (EASD)	Diabetes Care	164	The ADA and the EASD updated the previous consensus statements on managing hyperglycemia in T2DM in adults, published in 2006 and last updated in 2019. These include an additional focus on social determinants of health, the health care system, and physical activity behaviors, including sleep and weight management, as part of the holistic approach to diabetes management. The outcomes of cardiovascular and kidney trials involving GLP-1 RA contribute to shaping more comprehensive guidelines for cardiorenal protection in individuals with diabetes who face a heightened risk of cardiorenal complications.	Article
Cusi et al., 2022 [26]	American Association of Clinical Endocrinology Clinical Practice Guideline for the Diagnosis and Management of Nonalcoholic Fatty Liver Disease in Primary Care and Endocrinology Clinical Settings: Co-Sponsored by the American Association for the Study of Liver Diseases (AASLD)	Endocrine Practice	150	This guideline includes recommendations for diagnosing and managing persons with NAFLD, closely linked to obesity and T2DM. The paper suggests that management strategies should incorporate lifestyle modifications to create an energy deficit to facilitate weight loss. Additionally, it recommends weight loss medications, specifically glucagon-like peptide-1 receptor agonists, and bariatric surgery for individuals with obesity. Furthermore, the paper proposes exploring the potential use of certain diabetes medications, including pioglitazone and glucagon-like peptide-1 receptor agonists, for individuals with T2DM and NASH.	Article
American Diabetes Association Professional Practice Committee, 2022 [24]	Chronic Kidney Disease and Risk Management: Standards of Medical Care in Diabetes-2022	Diabetes Care	143	This paper includes the ADA’s current recommendations for diabetes care, treatment goals and guidelines, and quality of care evaluation tools. This paper states that semaglutide does not require dose adjustment for a reduced estimated glomerular filtration rate.	Article
Brown et al., 2021 [32]	SGLT2 inhibitors and GLP-1 receptor agonists: established and emerging indications	The Lancet	139	SGLT2 inhibitors and GLP-1 receptor agonists are employed as therapeutic options for individuals with T2DM to lower blood glucose levels, and they offer supplementary advantages such as weight loss and decreased blood pressure. Findings from CV outcome trials have underscored that these medications provide protective benefits against major CV disease in those with established ASCVD, reduce the risk of admission to hospital for heart failure, and reduce mortality.	Review

Discussion

**Figure 14:**
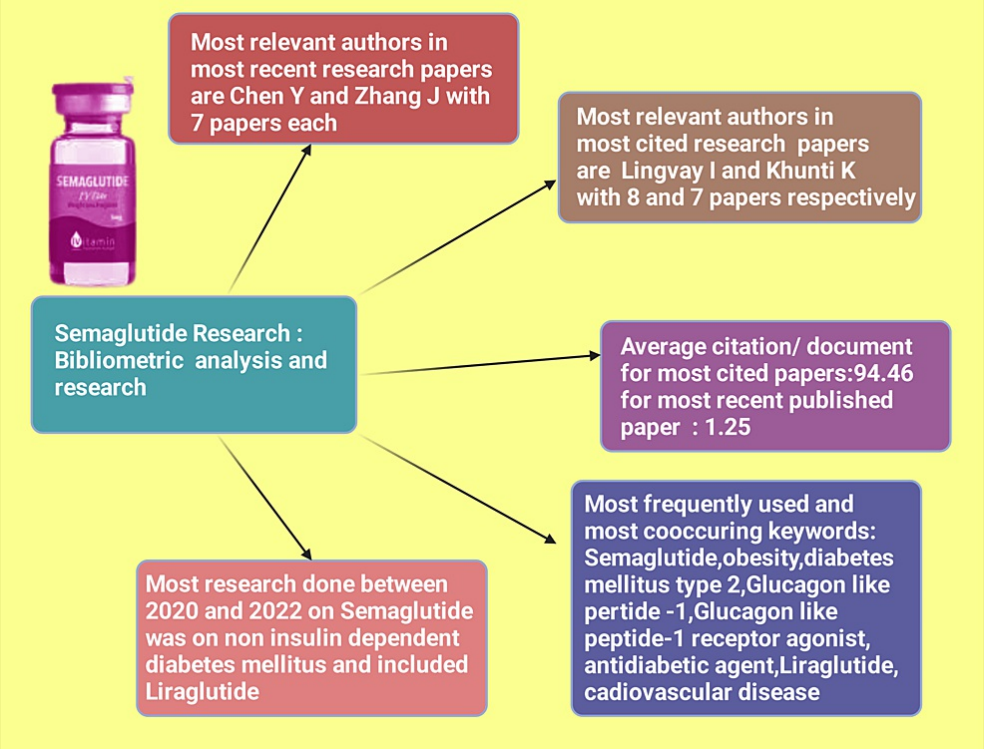
Displaying the schematic representation of the authors most cited and recent research articles on semaglutide, as well as the most frequently used keywords in this field of research Notes: This figure has been drawn with the premium version of BioRender (https://biorender.com/) with license number YW25U8FV00. Image credit: Rahnuma Ahmad

There is an increasing trend in the number of research papers from 2014 to 2022, with a peak in 2022 (Figure 2). The most relevant authors in the most recent semaglutide research papers are Chen and Zhang, with seven papers each. The most pertinent authors in the most cited research papers on semaglutide are Lingvay and Khunti, with eight and seven papers, respectively. The average citation per document is 94.46 for the most cited documents whereas 1.25 for the most recent published documents. The most frequently used keywords in the most cited papers and keywords identified in cooccurrence analysis in the most recent articles are semaglutide, obesity, diabetes mellitus type 2, glucagon-like peptide-1, glucagon-like peptide-1 receptor agonist, antidiabetic agent, liraglutide, and cardiovascular disease. No collaboration between any country is found in any research paper. Trend topic analysis suggests that most of the research between 2020 and 2022 on semaglutide was done on non-insulin-dependent diabetes mellitus and included data on another drug, liraglutide (Figure 14).

Until now, no bibliometric analysis has been done on semaglutide; however, two have been identified on GLP-1 RA [38,39]. One of these articles was related to the role of GLP-1 RA in renal disease [38], whereas another was related to its cardiovascular effects [39]. Both the research papers have identified “Diabetes, Obesity and Metabolism” as the most relevant source, which aligns with our results. Our analysis also determined that the most pertinent source is “Diabetes, Obesity and Metabolism” for the most recent and most cited research papers on semaglutide. However, the other findings are not the same, possibly due to the difference in the selected time and the database used for data collection. Both used the Web of Science database here, as we used Scopus. Also, these studies focused on all the research papers related to GLP-1 R, whereas we only focused on one drug of this group.

**Figure 15:**
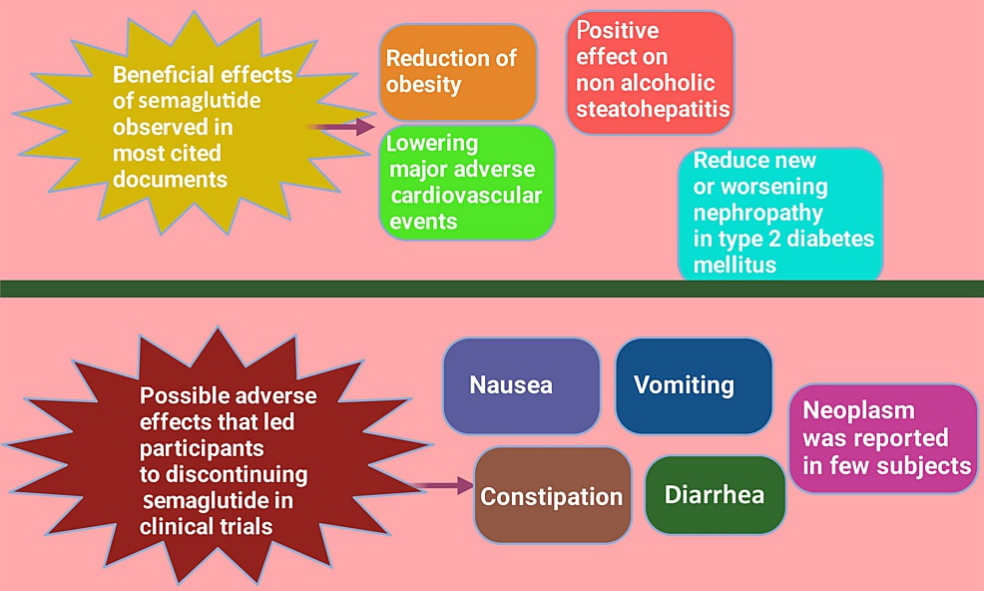
Depicting the possible health benefits of semaglutide as observed in the most sited documents in the field of semaglutide research and the adverse effects that led to the discontinuation of semaglutide by participants in clinical trials Notes: This figure has been drawn with the premium version of BioRender (https://biorender.com/) with license number TP25U8QYX6. Image credit: Rahnuma Ahmad

**Figure 16:**
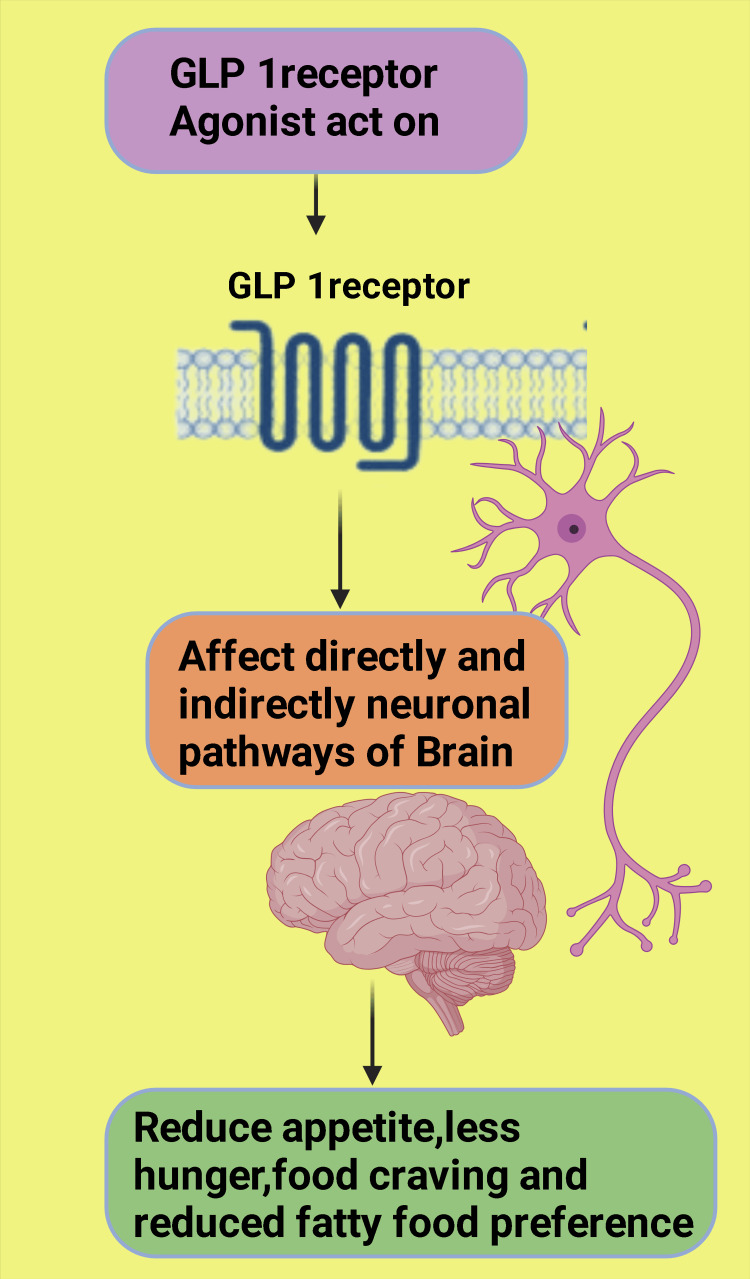
Illustrating the effect of GLP-1 receptor agonist on neural pathways leading to reduced appetite and food craving and decrease in affinity toward high-fat-containing food Notes: This figure has been drawn with the premium version of BioRender (https://biorender.com/) with license number OE25U8EFVO. Image credit: Rahnuma Ahmad GLP-1: glucagon-like peptide-1

The most cited documents, including clinical trials, reviews, and guidelines by the ADA, EASD, and American Association of Clinical Endocrinology Clinical Practice Guideline for the Diagnosis and Management of Nonalcoholic Fatty Liver Disease, suggest that semaglutide is effective in reducing obesity, major adverse cardiovascular events, non-alcoholic steatohepatitis, and the risk of new or worsening nephropathy in T2DM patients. No dose adjustment is required for semaglutide in chronic kidney disease, although renal function monitoring is recommended in patients reporting severe adverse gastrointestinal reactions. These findings align with a review done by Goldenberg and Steen in 2019 on semaglutide [40]. The main reasons for the subjects to discontinue participation in the clinical trials were gastrointestinal adverse events, such as nausea, constipation, diarrhea, and vomiting. Neoplasm is reported in a few subjects (Figure 15). GLP-1 RA use is not recommended in patients with a personal or family history of medullary thyroid carcinoma or those with multiple endocrine neoplasia syndrome type 2. The same facts are also highlighted by a review done by Ryan [41]. The findings of one of the highly cited studies in our review indicate that semaglutide lowers body weight by directly interacting with diverse GLP-1 RA populations and strictly and indirectly affecting the activity of neural pathways involved in food intake, reward, and energy expenditure in rats [37]. Similar findings are observed in human studies too. In a randomized, double-anonymized, placebo-controlled, two-period crossover study, 30 subjects with obesity were treated with once-weekly subcutaneous semaglutide for 12 weeks. The results showed that semaglutide reduced ad libitum energy intake significantly compared to the placebo, leading to a 24% reduction in total energy intake throughout the day. Also, participants reported less hunger and food cravings, better control of eating, and a reduced preference for high-fat foods (Figure 16) [4].

The reviews and clinical trials in the manuscript highlight the drug’s benefits; however, Novo Nordisk, the only company with FDA-approved semaglutide, has funded many of the clinical trials. Therefore, more data from unbiased sources is required to support the findings of these studies.

This study analyzed the most recent and the most cited research papers to find the latest and most relevant information. The study’s main limitation is that only one database was considered for the analysis, as it is impossible to merge the data from two different databases for analysis. Another limitation is that we reviewed the content and provided the summary for the most cited documents but not the most recent ones. Nevertheless, this study provides a comprehensive analysis of semaglutide research, and the highly cited papers offer essential insights into the use of semaglutide, a GLP-1 RA, in the management of diabetes, obesity, and related conditions, along with their potential benefits and adverse effects.

Semaglutide, a new drug approved by the FDA on 4 June 2021, has demonstrated effectiveness in managing weight and reducing hyperglycemia [2]. Clinical trials have revealed common side effects such as nausea, constipation, diarrhea, and vomiting. Caution is advised in its usage due to potential unknown long-term effects resulting from prolonged use [15,30]. There is a high possibility that many unknown adverse effects have not been detected yet; that possibly may come across after long-term consumption.

## Conclusions

Semaglutide, the only GLP-1 RA with oral formulation, has emerged as a frontrunner in this endeavor due to its remarkable efficacy in glycemic control and its potential to influence multiple facets of diabetes management. There is an increasing trend in the number of research papers from 2014 to 2022, peaking in 2022. The analysis highlighted that the most relevant authors in the most recent semaglutide research papers are Chen and Zhang and in the most cited research papers on semaglutide are Lingvay and Khunti. The most relevant source is “Diabetes, Obesity and Metabolism” for research papers on semaglutide. The most common keywords used in the research papers are semaglutide, obesity, T2DM, GLP-1, GLP-1 RA, antidiabetic agent, liraglutide, and cardiovascular disease. No collaboration between any country is found in any research paper. Trend topic analysis suggests that most of the research between 2020 and 2022 on semaglutide was done on non-insulin-dependent diabetes mellitus and included data on another drug, liraglutide. The most cited articles suggest that there is compelling data on the benefits of semaglutide in various clinical conditions, including obesity, cardiovascular events, diabetes management, and reverses steatohepatitis in persons with or without diabetes. Semaglutide can be used in chronic kidney disease without dose adjustment, although renal function monitoring in patients reporting severe adverse gastrointestinal reactions is recommended. However, GLP-1 RA should be used cautiously in the case of pancreatic disease and is contraindicated in patients with a history of medullary thyroid cancer. The common side effects reported are nausea, constipation, diarrhea, and vomiting. Neoplasia has also been reported in a few studies. Physicians are strongly advised to prescribe this medication with utmost caution, as long-term study results on its chronic use are not yet available, potentially revealing unknown adverse effects.

Clinical trials with larger sample sizes and long duration are still needed for more generalizable data and to rule out any side effects that are very rare or occur after the long-term use of the drug.
